# Protein Activity of the *Fusarium fujikuroi* Rhodopsins CarO and OpsA and Their Relation to Fungus–Plant Interaction

**DOI:** 10.3390/ijms19010215

**Published:** 2018-01-11

**Authors:** Alexander Adam, Stephan Deimel, Javier Pardo-Medina, Jorge García-Martínez, Tilen Konte, M. Carmen Limón, Javier Avalos, Ulrich Terpitz

**Affiliations:** 1 Department of Biotechnology and Biophysics, Biocenter, Julius Maximilian University of Würzburg, D-97074 Würzburg, Germany; alexander.adam@uni-wuerzburg.de (A.A.); stephan.deimel@stud-mail.uni-wuerzburg.de (S.D.); 2 Department of Genetics, Faculty of Biology, University of Seville, E-41012 Seville, Spain; jpardo6@us.es (J.P.-M.); jorgegarmar@gmail.com (J.G.-M.); carmenlimon@us.es (M.C.L.); avalos@us.es (J.A.); 3 Institute of Biochemistry, Faculty of Medicine, University of Ljubljana, Sl-1000 Ljubljana, Slovenia; tilen.konte@mf.uni-lj.si

**Keywords:** fungal rhodopsins, CarO, OpsA, *Fusarium fujikuroi*, *Oryza sativa*, rice–plant infection, green light perception, indole-3-acetic acid (IAA), bakanae, patch-clamp

## Abstract

Fungi possess diverse photosensory proteins that allow them to perceive different light wavelengths and to adapt to changing light conditions in their environment. The biological and physiological roles of the green light-sensing rhodopsins in fungi are not yet resolved. The rice plant pathogen *Fusarium fujikuroi* exhibits two different rhodopsins, CarO and OpsA. CarO was previously characterized as a light-driven proton pump. We further analyzed the pumping behavior of CarO by patch-clamp experiments. Our data show that CarO pumping activity is strongly augmented in the presence of the plant hormone indole-3-acetic acid and in sodium acetate, in a dose-dependent manner under slightly acidic conditions. By contrast, under these and other tested conditions, the *Neurospora* rhodopsin (NR)-like rhodopsin OpsA did not exhibit any pump activity. Basic local alignment search tool (BLAST) searches in the genomes of ascomycetes revealed the occurrence of rhodopsin-encoding genes mainly in phyto-associated or phytopathogenic fungi, suggesting a possible correlation of the presence of rhodopsins with fungal ecology. In accordance, rice plants infected with a CarO-deficient *F. fujikuroi* strain showed more severe bakanae symptoms than the reference strain, indicating a potential role of the CarO rhodopsin in the regulation of plant infection by this fungus.

## 1. Introduction

Fungi inhabit almost every ecological niche in all kind of ecosystems, where they have to face sudden changes in their growth conditions. Their survival capacity relies on the successful adaption of their physiology to diverse environmental scenarios, which requires efficient control of gene expression. A remarkable example is found in the colonization of host organisms, that in fungi implies differential regulation of many genes [1]. One of the most important physical parameters impacting on fungal life is sunlight [2]. Fungi possess various photosensory proteins that allow them to perceive different light wavelengths, and to adapt to changing light conditions [2,3]. While flavin-based proteins, such as those of the white collar, photolyase, vivid, and cryptochrome families perceive blue light, red light is sensed by phytochromes via biliverdin [4]. Many fungi are also equipped with membrane-embedded green light-sensing rhodopsins [5]. However, only a small number of these photoreceptors have been biophysically or biologically characterized [6,7,8,9].

Fungal rhodopsins belong to the super family of G-protein coupled receptors, and accordingly, consist of seven transmembrane helices (TMs). In ascomycetes, four clades of opsin-like proteins are distinguished [10], that are grouped around either HSP30 (heat shock protein 30 [11]), ORP-1 (opsin related protein 1 [12]), NOP-1 (*Neurospora* opsin protein 1 [13]), or CarO (from *Fusarium fujikuroi* CarO [14], also known as auxiliary ORP-like rhodopsins [15]). Only NOP-1-like and CarO-like opsins are microbial rhodopsins that exhibit the lysine residue required for covalent binding of the chromophore all-*trans*-retinal. In the rhodopsin protein, the retinal is located in an interior pocket surrounded by the TMs. Retinal isomerization from all-*trans* to 13-*cis* initiates a series of consecutive conformational alterations (photointermediates), which are correlated with protein function, known as photocycle. The NOP-1-related opsin clade can be subdivided into *Leptosphaeria* rhodopsin (LR)-like [9] and *Neurospora* rhodopsin (NR)-like [13] rhodopsins, due to their characteristic protein function [15]. NR-like fungal rhodopsins most likely provide sensory functions, and their photocycle is very slow [13,16]. By contrast, LR-like and CarO-like rhodopsins exhibit fast photocycles [8,14], and provide proton pumping activity [8,9,16]. A characteristic of CarO-like rhodopsins is the occurrence of a presumptive interaction site for a so far unknown transducer protein [7].

We investigate the rhodopsins of the fungus *F. fujikuroi*, an ascomycete that provokes bakanae disease in rice (*Oryza sativa*) plants [17]. The disease is accompanied by an increase in stem elongation, which is provoked by the plant hormone gibberellic acid (GA) produced by the fungus [18,19]. The infected plants become chlorotic (paler green tissues) and exhibit less leaves and internodes. In severe cases, even growth stagnation (stunted growth) can be observed [20], an effect provoked by the action of a cytotoxic secondary metabolite, fusaric acid, produced by the fungus [21,22]. *F. fujikuroi* possesses two genes coding for rhodopsins, *carO* and *opsA* [14,23]. The *carO* gene is linked to and co-regulated with genes coding for enzymes for retinal synthesis, whose expression is strongly induced by light, and the CarO protein is an effective proton pump highly expressed in light-exposed conidia, where it slows down their germination [8,24]. On the other hand, the *opsA* gene is only moderately upregulated by light, and the function of the OpsA rhodopsin is not known.

The prevalence of fungal rhodopsins in the genomes of phytopathogenic or phyto-associated fungi suggests a potential role in the host–fungus interaction [8]. Supporting this assumption, a recent analysis of the microbiome in the leaf environment of different plants revealed high abundance of rhodopsins in the phyllosphere [25]. Furthermore, the LR-like rhodopsin Sop1 from the fungus *Sclerotinia sclerotiorum* plays an essential role in the virulence of the fungus. Also, the two rhodopsins of the ascomycete *Alternaria brassicola* (gb|AB08921 and gb|AB06529) are upregulated in the plant host environment [26]. Similarly, the rhodopsin *ops3* (gb| Um04125) of the basidiomycete *Ustilago maydis* is upregulated during the infection process in corn plants [27]. In general, pH plays an important role on fungal virulence during the plant infection [28,29], which could be related with the involvement of proton pumping rhodopsins in fungal pathogenicity.

In the present study, we aim to improve our understanding of the biological role of the rhodopsins in *F. fujikuroi*. We expressed either CarO::YFP or OpsA::YFP in HEK293 cells, performed electrophysiology experiments, and observed that the presence of the auxin indole-3-acetic acid (IAA) and acetate increases the CarO pumping activity up to 10-fold, whereas OpsA did not show any net charge transfer under the tested conditions. We conducted a basic local alignment search tool (BLAST) search for the occurrence of fungal rhodopsins in 108 Ascomycota genomes, and observed that the majority of rhodopsin genes are found in phytopathogenic and phyto-associated fungi. Furthermore, we tested the ability of conidia from mutant *F. fujikuroi* strains to infect rice plants, and observed more severe bakanae symptoms in plants infected by null CarO mutants, compared to those infected by the reference strain, as indicated by their increased internodal length and reduced chlorophyll content. Based on RT-PCR experiments, we found no indication for any co-regulation of CarO and G proteins. Taken together, our data provide evidence on the participation of fungal rhodopsins in fungi-plant interactions and phytopathogenesis.

## 2. Results

### 2.1. CarO Pumping Activity Is Enhanced by Indole-3-Acetic Acid (IAA) and Acetate Whereas OpsA Does Not Exhibit Pumping Activity

**Figure 1:**
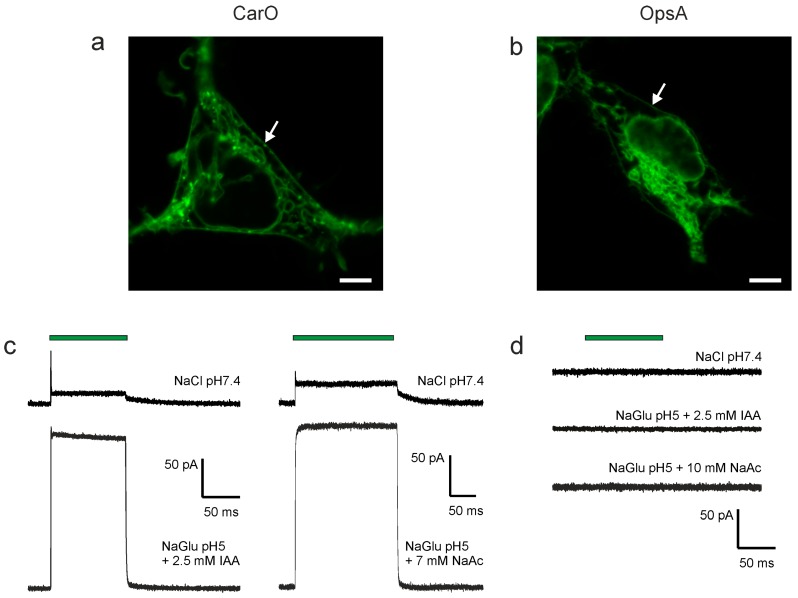
Microscopic and patch-clamp analysis of the fungal rhodopsins CarO and OpsA expressed in HEK293 cells. (**a**,**b**) Representative confocal laser scanning micrographs showing the localization of the fungal rhodopsins in the cells. Both rhodopsins are partly trafficked to the plasma membrane (indicated by **white arrows**), and thus, accessible to the patch-clamp pipette. Scale bars represent 5 µm. (**c**,**d**) Typical traces of either CarO (**c**) or OpsA (**d**) recorded in whole cell mode at 0 mV holding potential at intracellular pH 7.4 and diverse extracellular conditions, as indicated. Cells were illuminated with green light (532 nm DPSS laser), as illustrated by the green bar. The pump activity of CarO is clearly augmented by indole-3-acetic acid (IAA) and sodium acetate (NaAc) at pH 5, whereas for OpsA no electrogenic activity was detected under any of the tested conditions, suggesting a protein function unrelated with ion pumping.

During its pathogenic growth in the plant apoplast, *F. fujikuroi* faces different organic compounds, including weak organic acids (WOAs), and plant hormones like auxin [30]. Recently, we found that at low pH, the proton pump activity of CarO increased in gluconate solution [8]. Further investigation revealed that this supporting effect was mainly provoked by low concentrations of acetate ions used as counter ion for Mg^2+^ and Ca^2+^ in the gluconate solution, rather than by gluconate itself. As the pumping signal of CarO expressed in yeast cells was very low (Supplementary Figure S1), to further investigate this WOA effect, CarO::YFP and OpsA::YFP were expressed in HEK293 human cells (Figure 1a,b), and investigated with patch-clamp technique in a whole cell configuration. The rhodopsins were activated by illumination with green laser light (532 nm; 2–3 × 10^17^ photons s^−1^ mm^−1^). Under our experimental conditions, chloride channels are activated in HEK293 cells, interfering with the small pump signal [31]. Therefore, for a better signal-to-noise ratio, the WOA effect was measured in sodium gluconate-based (chloride-free) extracellular solution. Nevertheless, similar results were obtained in a NaCl-based solution.

Upon illumination under standard conditions (NaCl pH 7.4 bath solution) CarO exhibited the expected outward-directed signal [8], with a transient response that relaxed in the dark to the stationary level with a decay in a bi-exponential manner (Figure 1c). By contrast, in the presence of sodium acetate or the plant hormone IAA the activity of the proton pump CarO was substantially increased, and the ratio between peak and stationary current was clearly reduced (Figure 1c).

In the case of OpsA, no electrogenicity was found under all tested conditions, including those providing high pumping activity in CarO (Figure 1d), as expected from the assignation of OpsA to the group of NR-like rhodopsins [15]. This was further supported by sequence comparisons that revealed that OpsA is phylogenetically closer to NOP-1 than to CarO [23]. Like NOP-1 [32], these proteins are supposed to be non-pumping rhodopsins. Accordingly, NOP-1 was shown to exhibit a very slow photocycle, and did not provoke currents when expressed in neurons [16].

**Figure 2:**
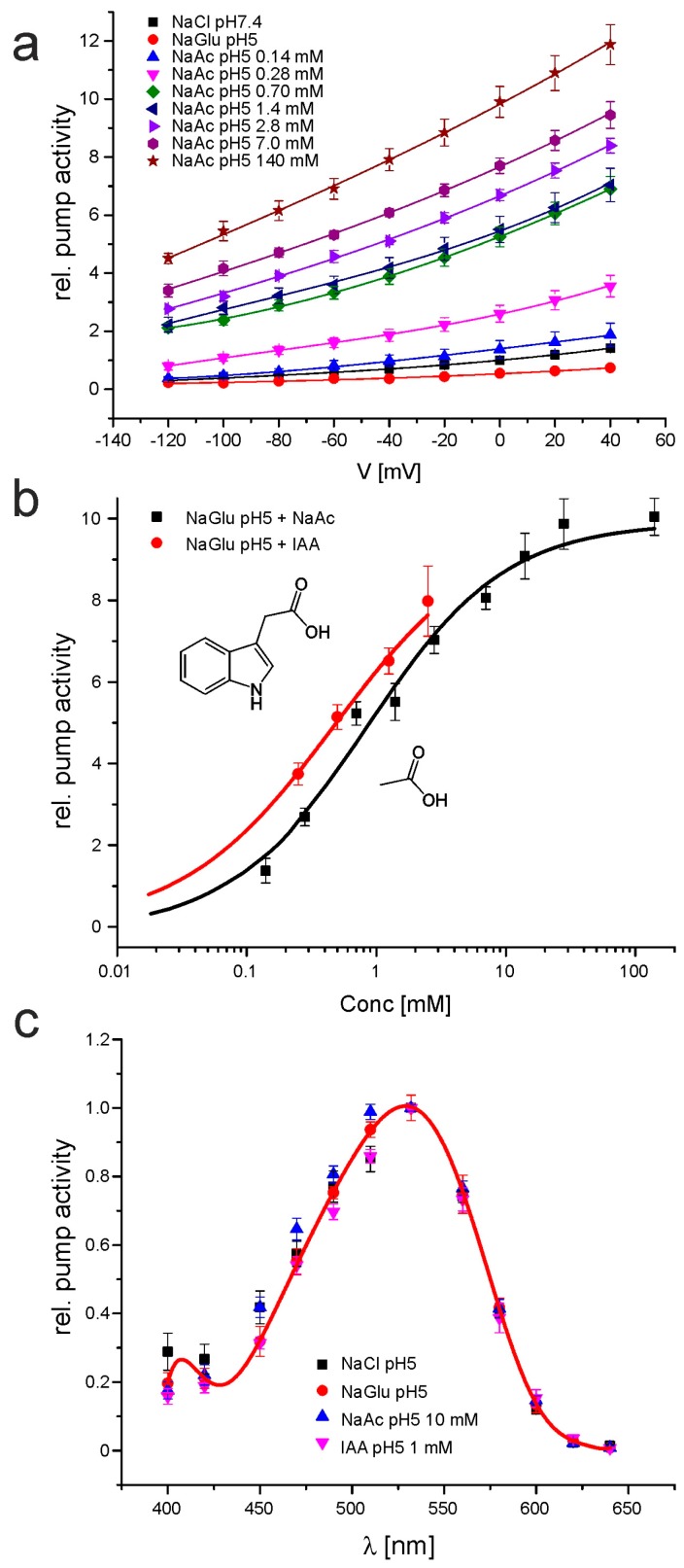
Patch-clamp analysis of the influence of sodium acetate and IAA in the pump activity of CarO. (**a**) Current–voltage relationship of the CarO pump activity, as indicated in sodium chloride pH 7.4, sodium gluconate pH 5, various concentration of sodium acetate in sodium gluconate pH 5, or only sodium acetate pH 5. Mean ± SEM of at least 5 cells are given. For better visualization, data were described by a cubic fit. (**b**) Dose–response relationship of CarO pump activity and IAA and sodium acetate dissolved in a bath solution of sodium gluconate pH 5. Data were described using a standard Hill equation. (**c**) Action spectrum of CarO as indicated in either NaCl pH 5, sodium gluconate (NaGlu) pH 5, sodium gluconate pH 5 + 10 mM sodium acetate, or sodium gluconate pH 5 + 1 mM IAA. For better visualization, the data points of NaCl pH 5 were fit using a polynomial fit. Note, that the spectrum is not influenced by the presence of the weak organic acids tested.

We further investigated the effect of WOA on the pump activity of CarO. When gluconate was gradually replaced by acetate, an increased pump activity of CarO was observed along the whole range of voltages, from −120 mV to +40 mV (Figure 2a). At the same time, the characteristic voltage dependency with higher activity at positive membrane potentials remained unaffected. A notable increase of pump activity became already observable in the two-digit micromolar range. Similar results were also obtained when gluconate was gradually replaced by IAA (Supplementary Figure S2). For better visualization, we plotted the dependence of pump activity at 0 mV membrane potential on the WOA concentration, and fit it with a Hill function (Figure 2b). The increase by IAA was in a similar range as by acetate, but it could not be analyzed in the full range of concentrations, due to its low solubility in water (Figure 2b). When acetate-activated CarO was faced with pH 9, a transient increment of the pump activity was observed, similar to the one recently reported in a gluconate solution ([8]; Supplementary Figure S3). The activity increased to a maximal 3.3-fold compared to that at 0.7 mM sodium acetate pH 5, and decreased within a few minutes to activities lower than those in NaCl pH 7.4. In bacteriorhodopsin, the pump activity did not increase in presence of WOAs, suggesting that this effect is specific for the CarO protein (Supplementary Figure S4).

In halorhodopsin and bacteriorhodopsin, the protonation of the Schiff base counter ion leads to a shift of the absorption spectrum [33,34]. In order to figure out if the presence of acetate or IAA might affect the protonation state, we recorded the action spectrum of CarO in sodium chloride pH 7.4, sodium gluconate pH 5, sodium gluconate supplemented with 1 mM IAA, or sodium gluconate supplemented with 10 mM sodium acetate. For all conditions, maximal pump activity was observed at 532 nm, and all data were in a similar range, suggesting unchanged protonation state of the counter ion of the Schiff base (Figure 2c).

### 2.2. Fungal Rhodopsins Are Predominant in Phyto-associated Fungi

**Figure 3:**
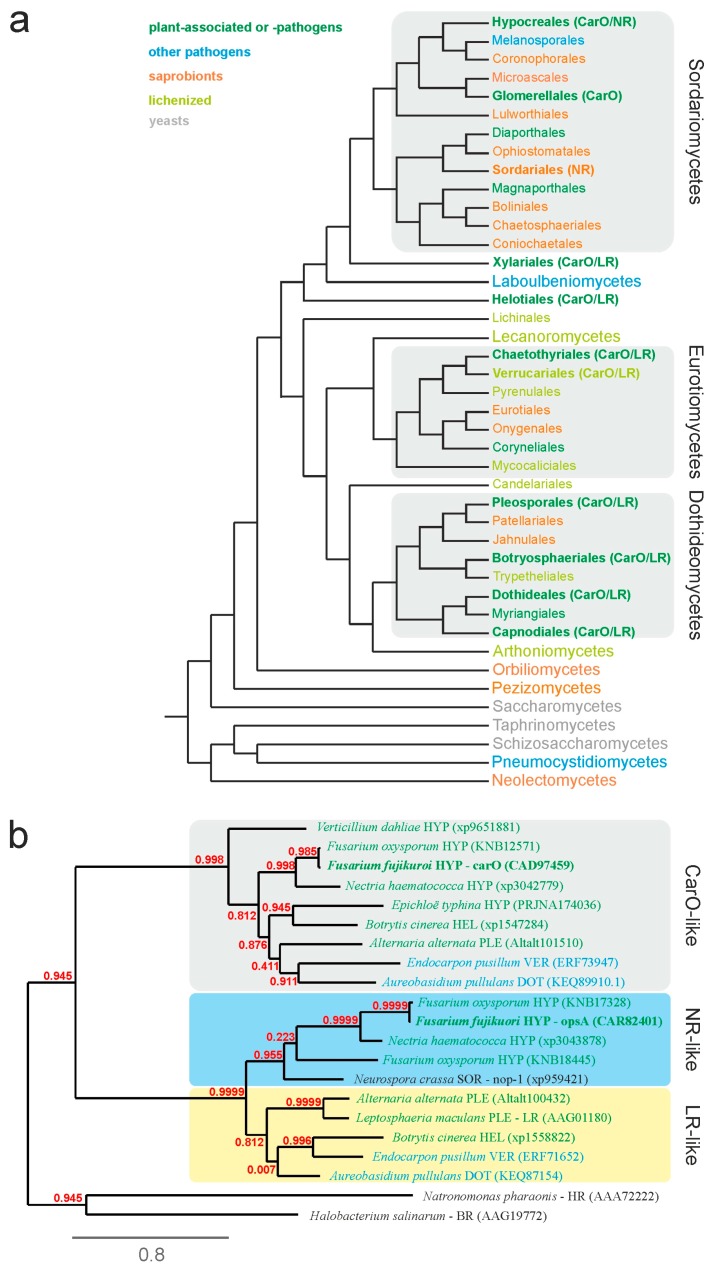
Phylogenetic analysis of ascomycetes and fungal rhodopsins. (**a**) Phylogenetic tree of the phylum Ascomycota. For each order, the typical ecological niche is indicated by the font color (green: phyto-associated or phytopathogenic fungi; blue: other pathogens; orange: saprobionts or wood inhabiting fungi; pale green: lichenized fungi; grey: yeasts). Orders that possess rhodopsins are printed in bold font, and the classification of the fungal rhodopsin is given in brackets. Three different classes of fungal rhodopsins are distinguished, NR-like (from *N. crassa*), LR-like (from *L. nodorum*, proton pumps), and CarO-like (auxiliary ORP-like rhodopsin, proton pumps with a putative capacity to interact with other proteins). NR-like and LR-like rhodopsins are merged in the clade of NOP-1 like opsins. Many phytopathogenic and phyto-associated fungi possess genes coding for rhodopsins, while most saprophytic fungi do not, indicating a potential role for these green light-sensing photoreceptors in fungus–plant interaction. A complete list with gene accession numbers is given in Supplementary Table S1. (**b**) Phylogenetic tree of fungal rhodopsins from selected species from Hypocreales (HYP), Helotiales (HEL), Pleosporales (PLE), Verrucariales (VER), Dothideales (DOT), and Sordariales (SOR), as indicated after the species name. Data in brackets represent gene database accession numbers. The tree that was rooted to the microbial halorhodopsin (HR) from *Natronomonas pharaonis*, and bacteriorhodopsin (BR) from *Halobacterium salinarum*, shows the phylogenetic relationships of fungal rhodopsins, including OpsA and CarO from *F. fujikuroi*. Branch support values are given in red, and branch lengths are as indicated by the bar. A complete tree is given in Supplementary Figure S4).

In our previous study, we found that rhodopsin genes are recurrent in the genomes of phytopathogenic fungi [8]. In order to find out if the presence of rhodopsins is more related to the phylogeny of the respective species than to its ecology, we analyzed the occurrence of rhodopsins in different fungal species by BLAST searches of their genome sequences (Supplementary Table S1). For this investigation, we focused our attention on the set of species used in a recent evolutionary analysis of fungal effector proteins [35], with some additions, arranged according to a former 6-gene maximum-likelihood phylogeny of Ascomycota [36]. Our phylogenetic analysis (Figure 3a) revealed that these photoreceptors are not present in all taxa. Out of 108 analyzed species from 42 orders, rhodopsins were only found in 38 fungal genomes. Interestingly, 74% of the species containing rhodopsins are either phytopathogens or phyto-associated (28 species). In accordance, rhodopsins were found in 9 out of 13 orders that include mainly phytopathogenic or phyto-associated fungi, while only in 1 out of 15 orders that include mainly wood inhabiting or saprophytic fungi.

As indicated above, the rhodopsins have been classified in three types, NR-like rhodopsins, with slow photocycle and presumable photosensory functions, and CarO-like and LR-like rhodopsins, with fast photocycles and proton pumping activities [15]. Though functionally distinct, LR-like and NR-like rhodopsins are clearly distinguished from the CarO-like rhodopsins, phylogenetically very closely related, and merge in one clade that was recently denoted as NOP1-like opsins [10]. We compared the amino acid sequences of the rhodopsins from the 38 fungi through different bioinformatic tools (see Experimental Procedures), and we used the generated data to construct a phylogenetic tree (Figure 3b, Supplementary Figure S4). The distribution of the rhodopsins in the tree supports the above-mentioned classification. Strikingly, no fungus was found to possess all three types of rhodopsins. By contrast, some fungi possess several copies or variations of the same type of rhodopsins. For example, the plant pathogen *Fusarium oxysporum* (Hypocreales), exhibits two different NR-like rhodopsins, while the human-pathogenic fungus *Coniosporium epidermidis* (Chaetothyriales) possesses two different CarO-like rhodopsins.

### 2.3. Bakanae Symptoms of Rice Plants Are Affected by the Null CarO Mutation in the Infective Fungus

Among the investigated species, CarO-like rhodopsins are spread in different taxonomic groups, but they are only present in phytopathogenic, human pathogenic, or rock-inhabiting species. However, rhodopsins are not found in all phytopathogenic fungi, e.g., they are absent in the rice pathogen *Magnaporthe grisea*. NR-like rhodopsins seem to be unique of the Sordariomycetes, while the combination of CarO-like and LR-like rhodopsins are mainly found in Eurotiomycetes, Dothideomycetes, and Helotiales. In the Sordariomycetes, only the Hypocreales and Glomerellales, both encompassing phytopathogenic fungi, exhibit CarO-like rhodopsins (Figure 3a, Supplementary Table S1). Overall, the presence of rhodopsins in mainly phyto-associated orders, independently of their phylogeny, suggests a positive selection for rhodopsins in plant environments.

**Figure 4:**
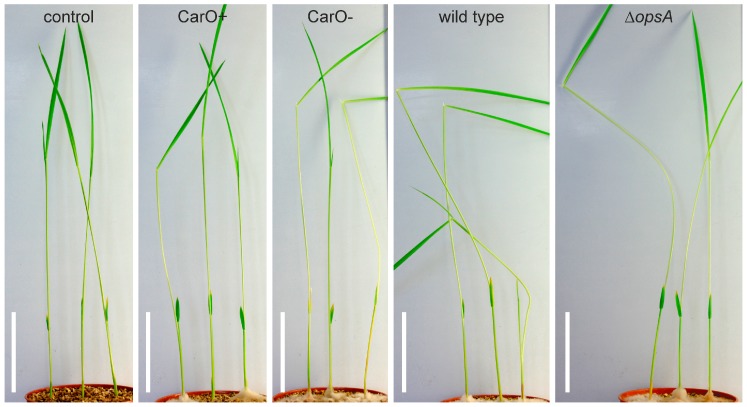
Rice plant infection with rhodopsin-deficient *F. fujikuroi* strains. Analysis of potential effects of the rhodopsins OpsA and CarO on the virulence of *F. fujikuroi* in rice plants (cultivar Sendra). Representative images of 13-day-old rice plants 10 days after infection with conidia from *F. fujikuroi*, strains or water (non-infected control), as indicated, are shown. Note that the plant shape varies even under the same conditions between the individuals. Therefore, to quantify the bakanae symptoms, the length of the first internode and the chlorophyll/carotenoid content were chosen to estimate disease severity (see Figure 5). White bars represent 5 cm.

**Figure 5:**
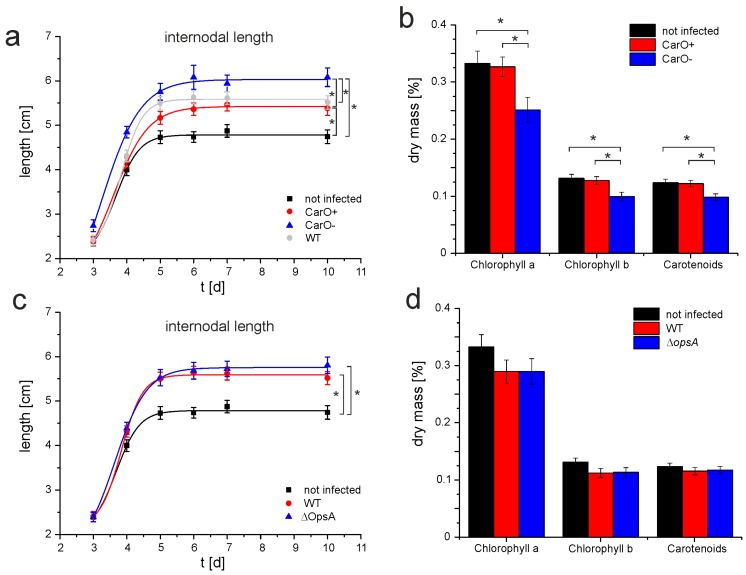
Influence of fungal infection on internodal length and chlorophyll content. (**a**,**c**) Mean length and standard error (data pooled from four independent experiments with 15 or 12 seedlings each for infected and control plants, respectively) of the first internode of rice plants. (**a**) Rice plants were either not infected (black, *n* = 42) or infected with the CarO^+^ (red, *n* = 58), the CarO^−^ (blue, *n* = 57) or wild type (gray, *n* = 56) strains. (**c**) Rice plants were either not infected (black, *n* = 42) or infected with wild type (red, *n* = 56) or the *opsA* deletion mutant (blue, *n* = 56) strains. (**b**,**d**) Chlorophyll and carotenoid extraction from rice plants 8 days after infection. Data are mean values and SEM of at least 12 trials out of 3 independent test series. (**b**) Rice plants were either not infected (black, *n* = 12) or infected with CarO^+^ (red, *n* = 15) or CarO^−^ (blue, *n* = 14) conidia. (**d**) Rice plants were either not infected (black, *n* = 12) or infected with wild type (red, *n* = 14) or *opsA* deletion (blue, *n* = 15) conidia. Differences in internodal length and chlorophyll/carotenoid content were tested for significance in a two-tailed student *t*-test. Significant distinctions are highlighted by an asterisk.

As rhodopsins are abundant in phyto-associated fungi, we aimed to find out if *F. fujikuroi* strains that are deficient in either OpsA [23] or CarO [14] would exhibit a distinct phenotype when infecting rice plants. In this experiment, previously established fungal strains were used, namely wild type (FKMC1995 *Fusarium fujikuroi* (*G. fujikuroi* mating population C)); Δ*opsA* (mutant FKMC1995 strain with deleted rhodopsin gene *opsA* [23]), CarO^−^ (mutant FKMC1995 strain with disrupted rhodopsin gene *carO* (shift in open reading) [14]), and CarO^+^ (isogenic reference of the CarO^−^ strain with intact rhodopsin gene *carO*). To analyze bakanae symptoms, we infected two-day old rice germlings (cultivar Sendra) with fresh conidia (50 conidia/rice seed) of the respective *F. fujikuroi* strain or water (control) under green light and grew the developing rice plants in vermiculite pots in light–dark cycles at 28 °C. All plants that were infected with fungi exhibited clear bakanae symptoms (Figure 4). Nevertheless, for each fungal strain, we noticed high variability in the morphology of the plant replicates. Thus, statistical analysis was required, and the symptoms of bakanae were assessed by differences of internodal length and changes in the plant chlorophyll content (Figure 5). While internodes of uninfected control plants exhibited after 10 days a mean length of 4.74 cm (SEM 0.15, *n* = 42), all infected plants exhibited longer internodes (CarO^+^ 5.38 cm, SEM 0.16, *n* = 58; CarO^−^ 6.08 cm, SEM 0.21, *n* = 57, Figure 5a; WT 5.53 cm, SEM 0.15, *n* = 56; ΔOpsA 5.81 cm, SEM 0.18, *n* = 56, Figure 5c). The CarO-deficient mutant produced a significant (*p* < 0.05; two tailed student *t*-test) increase in the internodal length of the rice plant (Figure 5a), compared to its reference strain CarO^+^, and the wild type, thus showing more severe symptoms of the disease. Also, the plants infected by the CarO-deficient mutant exhibited a paler pigmentation, and their content of chlorophylls/carotenoids was significantly reduced in comparison to control plants, or to those infected with the CarO^+^ reference (Figure 5b). Moreover, colonization of the rice seeds by fungal mycelium was faster with conidia of the CarO-deficient strain than with those of the CarO^+^ control (data not shown). By contrast, we did not detect any significant difference between the chlorophylls /carotenoids content of plants infected with wild type conidia, and that of plants infected with Δ*opsA* conidia, though both samples showed lowered content of these pigments when compared to non-infected plants. These data suggest that OpsA does not influence the bakanae symptoms (Figure 5c,d).

Addition of gibberellin GA3 to the nutrient solution results in an elongation of the rice plants (data not shown). As the infection with the CarO^−^ strain induces stem elongation, we aimed to find out if gibberellic acid (GA) synthesis was enhanced in the absence of CarO. However, the *carO*^−^ mutation did not affect the gibberellin production under laboratory conditions (Supplementary Figure S5). Similarly, OpsA does not affect gibberellin production [23]. These data suggest that the observed differences in bakanae symptoms might be due to the differential capacity of both strains to grow in the plant. In support to this conclusion, a higher number of mycelial colonies were able to grow from stems of infected rice plants when the infection was achieved with the CarO deficient mutant than with the CarO^+^ control strain (Supplementary Figure S6).

### 2.4. Light Exerts Minor Influence on mRNA Levels for Genes of G Proteins

**Figure 6:**
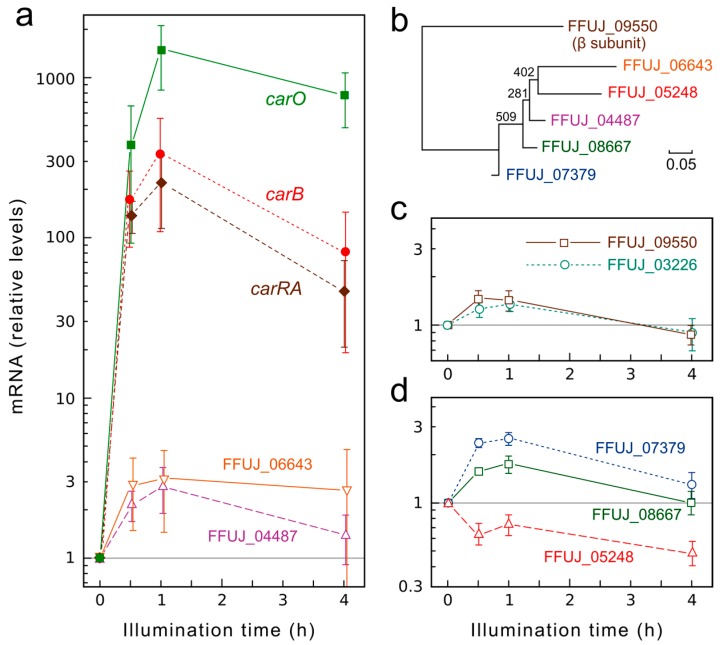
Effect of illumination on transcript levels for the genes *carRA*, *carB*, and *carO* and the seven genes for G proteins in the *F. fujikuroi* genome. (**a**,**c**,**d**) Real-time RT-PCR analyses of the indicated genes in total RNA samples from the wild type FKMC1995. The cultures were grown for three days in the dark before illumination for 30 min, 1 h, or 4 h. Relative levels are referred to the value of the wild type in the dark. Data show means and standard errors of the mean from three biological replicates. The data are represented in different graphs to avoid overlapping. (**b**) Phylogram of the sequences of the five Gα proteins (FUJ_06643, 04487, 07379, 08667 and 05248). The Gβ protein (FUJ_09550) was used as outgroup. The Gγ protein (FUJ_09550) was not included in the phylogram because of its small size (93 residues). The scale indicates the proportion of changes. Numbers in nodes indicate bootstrap values.

The finding of a presumptive interaction site for a putative transducer protein in the CarO-like protein of *Phaeosphaeria nodorum* [7] suggests that CarO might interact with a regulatory partner. Because of the participation of heterotrimeric G complexes in signal transduction from different animal opsins, we postulate that CarO might interact with a specific Gα protein. Considering that CarO expression is strongly light dependent, we might expect some level of co-regulation with its putative transducing partner. To check this hypothesis, the genome of *F. fujikuroi* was screened for the presence of genes for putative G proteins, based on a previous scrutiny carried out in *Gibberella zeae* (*Fusarium graminearum*) [37]. We found in *F. fujikuroi* five genes for putative Gα subunits (FUJ_06643, 443 aa; FFUJ_04487, 355 aa; FFUJ_07379, 354 aa; FFUJ_08667, 353 aa; FFUJ_05248, 636 aa), and single genes for a Gβ subunit (FUJ_09550, 359 aa) and a Gγ subunit (FFUJ_03226, 93 aa). A phylogram of the Gα and Gβ sequences confirmed that the five α subunits are more closely related to each other than to the β subunit (Figure 6b).

To check a possible co-regulation with *carO*, we investigated the effect of illumination on the transcript levels of the seven genes for G proteins in comparison to those of the gene *carO* and the structural genes of the carotenoid pathway *carRA* and *carB*, linked to *carO* in a co-regulated cluster [38]. As expected, the mRNA levels for these genes increased rapidly following light onset to reach 200–300-fold in the case of *carRA* and *carB*, and more than 1000-fold in the case of *carO* (Figure 6a). By contrast, the genes for G proteins were very moderately affected by light, with maximal variations of only 3-fold (Figure 6a–d). Such changes are very small compared to the strong photoinduction exhibited by *carO*, suggesting lack of regulatory connections.

Recently the gene expression profile of *F. fujikuroi* strain FKMC1995 in vitro and in planta was published [39]. We compared the expression levels of the above-mentioned proteins at 6 mM glutamine (22.7 mM in our experiments), with the in planta conditions (Supplementary Table S2). In accordance with the RT-PCR data we found the level of G proteins to be almost unchanged (ratios between 0.7 and 1.4), while *carO* (3.3) and *carB* (2.7) were upregulated in the plant environment. In contrast, *carRA* was almost unchanged (1.1) and *carX* (0.3) even downregulated in planta.

## 3. Discussion

Despite the efforts dedicated to the detailed characterization of several fungal rhodopsins [7,8,9,13,14,40], the biological role and impact of these proteins in filamentous fungi is still elusive. In previous investigations, the phenotypic differences of the rhodopsin-deficient mutants compared to the wild type were absent or hardly detectable [13,14,23,40]. Strikingly, in our recent study, CarO was found to slow down the germination of *F. fujikuroi* conidia in light [8]—the first phenotype associated with a fungal rhodopsin. A recent, detailed analysis of NOP-1 from *N. crassa* now revealed a clear phenotype of the fungal rhodopsin in the regulation of the sexual–asexual switch [10]. However, the analyses were performed using cultures grown in defined nutrient media under standard laboratory conditions, in the absence of a host plant.

To our knowledge, there is yet only one investigation dealing with the phenotype of rhodopsin-deficient mutants of phytopathogenic fungi in their host plant environment [41]. In this investigation, the LR-like rhodopsin Sop1 was shown to be essential for growth, sclerotial development, and full virulence of *S. sclerotiorum*. In accordance, our BLAST search for rhodopsins in a hundred sequenced fungal genomes (Figure 3; Supplementary Figure S5 and Supplementary Table S1) reveals that the majority of genes coding for fungal rhodopsins are found in phyto-associated or phytopathogenic fungi (fungi colonizing living plant tissue), whereas only a small percentage of saprotrophic fungi (fungi decaying organic matter) exhibit such genes in their genomes. The number of phyto-associated fungi might be even bigger, as the ecology of many fungi is not well investigated, and might conceal some surprises, as happened recently with the discovery of an unknown phyto-associated lifestyle of *N. crassa* [42].

To figure out a potential role of NR-like and CarO-like rhodopsins in colonization of plants, we tested the ability of rhodopsin-deficient *F. fujikuroi* strains to colonize rice plants and to provoke the characteristic symptoms of the bakanae disease [17,20,21]. Recent RNA-seq data have shown that both rhodopsins are expressed in the plant environment [39] (Supplementary Table S2). According to this study, transcription of *carO* is only slightly (ratio 3.2), and *opsA* highly (ratio 28.6) upregulated in plants. In our experimental analysis, the extension of the plant shoot, measured as the length of first internode, and the content of chlorophyll and carotenoids, were chosen as indicators for infection severity. Indeed, our data show, for the first time, that a fungal rhodopsin of a phytopathogenic fungus affects the severity of rice plant infection (Figure 4 and Figure 5). When investigating the role of CarO and OpsA in the development of bakanae symptoms, the shoots between seed and first node are significantly enlarged in all infected plants, compared to uninfected control plants. Unexpectedly, the elongation of the shoot was significantly enhanced in plants infected with CarO^−^ strain, in comparison to those infected with the CarO^+^ or wild type strain. Moreover, the chlorophyll content in the CarO^−^ strain infected plants was lower than in those infected with the CarO^+^ strain, suggesting a more aggressive phytopathogenic phenotype in absence of the CarO rhodopsin. Similar symptoms could also be induced by treatment of plants with GA [20] and GA-producing *Fusarium* species are more efficient rice pathogens than those unable to produce GAs [43]. Moreover, comparison of pathogenic capacities of wild type *F. fujikuroi* and a GA-defective mutant on rice revealed an impaired ability of the mutant hyphae to penetrate the plant cells [44]. In our case, the amount of GAs produced in shake cultures of the CarO-deficient strain was similar to those produced by the control CarO^+^ strain (Supplementary Figure S6). We cannot exclude that the GA-production of *F. fujikuroi* in the plant environment differs, and might be influenced by CarO, a task to be investigated in the future. However, based on our data, the longer internodes in the CarO^−^-infected plants should be interpreted as a result of a higher amount of fungal biomass compared to those infected with the CarO^+^ control strain. Accordingly, the CarO deficient strain was found to be more effective in colonizing the plants (Supplementary Figure S7). Thus, one might conclude that the increased severity of bakanae symptoms in absence of CarO is caused by an increased virulence.

Fungal virulence is directly related to the speed of germination [45]. The faster the fungus invades the host tissue, the lower the risk for the conidia to be displaced or outcompeted by other fungi [46]. In this respect, it must be noted that under laboratory conditions, the conidia of the CarO^−^ mutant germinated earlier and faster than those of the CarO^+^ strain [24], whereas in later developmental stages, both strains exhibit a similar colony growth, i.e., mycelial development [8]. Therefore, the faster germination may not be sufficient to explain the observed increase in virulence. Recent findings about the role of rhodopsins suggest negative regulatory role under light, like CarO in conidia germination and NOP-1 in protoperithecial production in the sexual cycle [8,10]. It can be speculated that rhodopsin activity might also negatively regulate the virulence of *F. fujikuroi*. The molecular mechanisms underlying the observed effects will require further investigation. Also, the potential importance of carotenoid production for rhodopsin function has to be taken into account. In *F. fujikuroi*, the *carO* gene is located and co-regulated in a cluster with genes coding for enzymes involved in carotenoid synthesis, namely *carX*, *carRA*, and *carB*. Interestingly, in 8 fungi out of the 11 fungal species shown in Figure 3b, the *carO* counterpart was clustered with orthologs for these *car* genes (Supplementary Table S3). Recent RNA-seq data show that *carB* is upregulated, *carRA* almost unchanged, and *carX* downregulated during plant colonization. Thus, *F. fujikuroi* with deleted *car* genes should be considered in future pathogenesis studies in rice plants.

To date, the role of light in phytopathogenesis by fungi has received very limited attention. Nevertheless, some investigations have revealed connections between fungal light perception and pathogenicity. In *Magnaporthe oryzae*, pre-illumination of conidia for one hour did not affect spore germination, but stimulated their chemically assayed superoxide production, resulting in spores more tolerant to toxic diffusate of rice leaf compared to those that were non-illuminated [47]. In the grey mold *Botrytis cinerea*, the proteins of the white collar photoreceptor complex are required for coping with excessive light or oxidative stress, and also to achieve full virulence [48], and in *Colletotrichum acutatum*, the light quality influences the virulence of the fungus [49]. Remarkably, the fungus *Diplodia mutila* leaves its endophytic niche to become a pathogen in response to high–intense light. This is most likely triggered by light-induced production of H_2_O_2_ by the fungus, which results in hypersensitivity, cell death, and tissue necrosis in the infected palm [50]. In this respect, it is of interest that the CarO protein of the closely related fungus *F. graminearum* was recently shown to be highly upregulated after initiation of the sexual cycle [10]. Besides that, recent investigations in *N. crassa* and *S. sclerotiorum* suggest a role of rhodopsins in oxidative and osmotic stress [10,41].

Another environmental factor that plays a critical role during infection processes in phytopathogenic fungi is pH [29]. Because of its effective proton pumping activity, CarO may contribute to local pH changes in infected tissues. Fungi, in general, use proton gradients for the signaling and transport of substances through membranes [51]. A green light-driven proton pump could thus play a role as an energy saving mechanism. In accordance, our microscopic data show that the CarO protein is expressed in light-exposed hyphae [8]. The *carO* gene is actively induced by light in older mycelia (four day old submerged cultures [14]), and although the physiological conditions in the plant may be very different than under ex planta laboratory conditions, the data suggest that the gene is also induced by light in the plant, a fact that could be favored by the higher efficiency of the rhodopsin under green light. By contrast, the OpsA^−^ mutant did not exhibit altered bakanae symptoms in comparison to the wild type (Figure 5c,d). OpsA is a NR-like rhodopsin, only present in the Sordariamycetes, and similar to NOP-1 of *N. crassa*, a rhodopsin with no electrogenicity [16] and with a putative sensory function. In accordance, we could not observe any pumping activity of OpsA, though we tested it under several experimental conditions (Figure 1d). Nevertheless, we cannot exclude that OpsA might behave differently in its native environment, and the localization and biological role of OpsA will be matter of future investigations.

The strong increment in pump activity of the outward-directed proton pump CarO, in response to the plant hormone IAA and to acetate at moderate acid pH (Figure 2a–c), is of high interest, because of the predictable implications in the fungus–plant interaction. In principle, under such conditions, we would expect a reduction of pump activity due to the increased proton gradient. A number of different ion species, such as sodium, chloride, or imidazole, are known to be pumped or guided by microbial rhodopsins [52]. Therefore, we may speculate that the ion species transported by CarO might be not restricted to protons. Acetate is a small anion, and thus, theoretically it could be considered as a potentially pumped species. This argumentation is refuted by our comparative experiments with IAA, which is too big to pass through the ca 0.6 nm pore of a microbial rhodopsin [53,54], and shows similar results as with acetate (Figure 2b). IAA and acetate are capable of diffusing in the undissociated form through the membrane with permeability coefficients of 6.9 × 10^−3^ cm s^−1^ [55] and 2.3 × 10^−4^ cm s^−1^ [56], respectively. Intracellularly, the compounds can dissociate, as indicated by the capacity of acetate to symmetrically acidify the cytosol in frog oocytes [57,58]. Suchlike perfusion through the membranes is the method by which IAA is distributed in the growing plant. However, in our patch-clamp experiments, the cytosolic conditions are well defined, as the cytosol is rapidly replaced by the pipette solution [59]. We therefore assume that the increase of pumping activity is not due to a potential uncoupling mechanism of acetate or IAA. Instead, it is more likely that the acetate group interacts with the free hydrogen in the pump pore, and consequently enables faster release of protons, and therefore, speeds up the pumping activity. A similar behavior was formerly described for other microbial rhodopsins [60], but we could not observe a comparable response of bacteriorhodopsin to acetate or IAA at pH 5 (Supplementary Figure S4). However, we found a supporting effect of WOAs for other fungal rhodopsins (unpublished data), indicating that this effect is of biological relevance in fungal rhodopsins. This could especially be the case in the plant environment, where IAA and acetate and other WOAs are present in the plant sap [30]. IAA was recently shown to be produced by *Fusarium* species [61], and to play a role in the bakanae symptoms evoked in rice plants by *Fusarium proliferatum* [62].

It has to be taken into account that our electrophysiological investigation was performed in rhodopsins expressed in mammalian cells. Though this is a common procedure for characterization of microbial rhodopsins, it must be taken into account that the protein might behave differently in its native environment, e.g., due to the absence of potential interacting/transducing proteins. We considered the possible participation of a G protein as a CarO regulatory partner, an interaction solidly established in the case of the visual opsins in vertebrates, and some non-visual proteins in lower animals [63,64]. However, the apparent lack of strong regulation by light of any of the G protein genes of *F. fujikuroi* does not favor this hypothesis, but it cannot be discarded. More experiments are needed to confirm if CarO-like rhodopsins only have proton-pumping activity, or whether they also have a signal-transducing activity through the interaction with a G protein or with a membrane-embedded regulatory protein.

The functions of rhodopsins in fungi are puzzling and fascinating biological issues. Our data suggest that the two *F. fujikuroi* rhodopsins play very different roles, reflected by their distinct biochemical properties and regulatory patterns. In the case of CarO, we could show for the first time that a rhodopsin influences the behavior of a fungus during plant infection by reducing the severity of disease symptoms. Although its mechanism of action and its participation in other cellular processes remain to be elucidated, we may speculate that green light is interpreted by the fungus as a signal to attenuate the strength of the pathogenic colonization of the plant. The ways in which the photobiochemical activities of different fungal rhodopsins influence the physiology of these organisms, and their ability to survive in their natural environment, will be the aim of further studies.

## 4. Materials and Methods

### 4.1. Fungal Strains and Culture Conditions

All fungal strains used in this study are derivatives of *Fusarium fujikuroi* FKMC1995. Phenotypic analyses of CarO were performed with the strains SF100 (CarO^−^; transformant 4 R3) and SF101 (CarO^+^ control; transformant 4 R2), both derived from a former transformant with a *carO*^+^ *carO*^−^ duplication generated by homologous integration with a *carO*^−^ plasmid [14]. SF100 contains a disrupted CarO allele, whereas SF101 is a revertant strain from the same transformant exhibiting an intact CarO allele. Phenotypic analysis of OpsA was done with strain SF223 (Δ*opsA*; [23]) that is lacking the OpsA gene, and FKMC1995 wild type strain was used as control. Fluorescent strains WF2 (*F. fujikuroi* CarO::YFP [8]) was described before. Fungi were cultured in Dextrose-*Gibberella* (DG) minimal medium [65] where NaNO_3_ was replaced by 3 g L^−1^ glutamine (DG_gln_), or in potato dextrose agar (PDA). Fungi were grown in a Peltier-controlled incubator (Memmert IPP110) equipped with an appropriate light module. In addition, a customized illumination module consisting of 10 green high-power LEDs (205 lm, LUXEON Rebel LXML PM01, Lumileds; San Jose, CA, USA) controlled by a customized power supply was used for green light illumination of fungal cultures. Dark-grown cultures were protected against light by storing them in an opaque box inside the incubator.

### 4.2. Cell Cultures

Stable cell lines of Flp-In™ 293 T-REx (Invitrogen, Carlsbad, CA, USA) expressing either CarO::YFP [8] or OpsA::YFP (this study; implemented according to manufactures manual) were cultured at 37 °C and 5% CO_2_ in Dulbecco’s Modified Eagle Medium (DMEM) supplemented with fetal calf serum (10%), l-glutamine (2 mM), penicillin (100 U mL^−1^), streptomycin (100 µg mL^−1^), and as selection, antibiotics hygromycin B (100 µg mL^−1^) and blasticidin HCl (15 µg mL^−1^). For experiments, the cells were grown in a 6-well plate with 2 mL medium/well. Protein expression was induced by tetracycline (3 µg mL^−1^) for 12–16 h. Cells were treated with all-*trans*-retinal (1 µM) to ensure sufficient availability of chromophore for the tested rhodopsins.

### 4.3. Molecular Biology

For the construction of pcDNA5/FRT/TO-opsA::YFP, the cDNA of the *opsA* gene without stop codon and fused to *eyfp* with a 21-bp linker was synthesized (Gene Art, Life Technologies, Waltham, MA, USA) and delivered in the vector pMA-RQ. This 1.6 kb fragment was cut by *Xho*I and ligated to the pcDNA5/FRT/TO-backbone cut with *Xho*I, resulting in pcDNA/FRT/TO-opsA::YFP.

### 4.4. Patch-Clamp

Patch-clamp experiments were performed as described by García-Martínez et al. [8] with some modifications. For activation of the rhodopsins, a 532 nm DPSS laser (MGL-III-532, 150 mW, TTL 1 Hz–1 kHz; Changchun New Industries Optoelectronics, Changchun, China) was coupled into the fluorescence beam path of an inverse microscope (Axiovert 200, Zeiss, Jena, Germany). Pipettes (GB150F-8P, Scientific-Instruments, Hofheim, Germany) had a tip opening diameter of 1.5 mm and exhibited a resistance of 3–5 MΩ in standard bath solution. Whole cell currents were recorded with an Axopatch 200B amplifier coupled to a DigiData 1440 interface (Molecular Devices Corporation, Union City, NJ, USA), low-pass filtered at 5 kHz, and digitized at a sampling rate of 100 kHz with the software Clampex 10.3 (Molecular Devices Co.). Data analysis was performed using Clampfit 10.6 (Molecular Devices Co.) and Origin Pro 9.1G or 2016G (OriginLab Corporation, Northampton, MA, USA). The experiments were performed in a customized perfusion chamber offering convenient slow solution exchange with a flow rate of ca 2 mL min^−1^. Standard bath solution contained 140 mM NaCl, 2 mM MgCl_2_, 2 mM CaCl_2_, and 10 mM 4-(2-hydroxyethyl)-1-piperazineethanesulfonic acid (HEPES) pH 7.4. For pH screening, 2-(*N*-morpholino)ethanesulfonic acid (TRIS) or 2-(*N*-morpholino)ethanesulfonic acid (MES) was used instead of HEPES, and the pH of the bath solution was adjusted to 5 or 9 with HCl or NaOH. The influence of WOAs on the rhodopsins was analyzed in solution buffered with either 10 mM HEPES, TRIS, or MES. The gluconate solution contained 140 mM sodium gluconate, 2 mM magnesium gluconate, and 2 mM CaOH, the respective pH was adjusted with either gluconic acid or NaOH. The acetate solution consisted of 140 mM sodium acetate, 2 mM magnesium acetate, and 2 mM calcium acetate, and the respective pH was adjusted with either acetic acid or NaOH. Acetate solution was mixed with either standard bath solution or gluconate solution to yield the final concentration. IAA was dissolved in ethanol to a final concentration of 250 mM, and added directly to the respective physiological solution.

### 4.5. Fluorescence Microscopy

Conidia of *F. fujikuroi* were inoculated in DG_gln_-medium in 8-well Labtek II chambers which were treated 1 h with 0.5 M NaOH and coated with 0.01% poly-d-lysine (125 µL per well) for at least 2 h at RT. Conidia were fixed directly or germinated at 28 °C in presence of light (6 W m^−2^) for 15–18 h. Samples were fixed for 10 min in 1% formaldehyde in phosphate buffered saline (PBS, pH 7.4) and stored in PBS at 4 °C. Fluorescence microscopy was performed at a confocal laser scanning microscope (SP700, Zeiss, Jena, Germany). Images were processed with ZEN software (ZEN 2012, Zeiss, Jena, Germany) or Fiji [66] with ImageJ 1.50f [67].

### 4.6. Rice Plant Infection Assays

Rice seeds belong to the Japonica variety J. Sendra, used in different rice fields in Spain [68], kindly provided by the Federación de Arroceros de Sevilla (Isla Mayor, Sevilla, Spain). Rice seeds were sterilized in a desiccator for 15 h with chlorine gas (33 mL 25% sodium hypochlorite mixed with 2.6 mL 25% HCl) immediately before their use. Sterilized seeds were transferred to 96-well plates, and germinated in 350 µL/well 0.1× yeast peptone dextrose (YPD) medium for 48 h at 28 °C in 12/12 light–dark cycle (12 h light: 1 h 3 W m^−2^, 3 h 6 W m^−2^, 4 h 10 W m^−2^, 3 h 6 W m^−2^, 1 h 3 W m^−2^) and additionally illuminated with green light (see section about fungal strains and culture conditions). Only seedlings that had developed shoots/roots of 1–2 mm length were chosen for further experiments. Seedlings were washed with 1 mL H_2_O, transferred into 24-well plates on sterilized filter paper, and exposed to 500 µL H_2_O supplemented with 50 conidia freshly harvested from light-exposed cultures of the respective strain. Control plants were exposed only to H_2_O. Infection of the seeds occurred within 24 h under continuous green light (2 × 10^−15^ photons s^−1^ mm^−2^) and 12/12 light–dark cycles with white light (3 × 10^−15^ photons s^−1^ mm^−2^). The green light was used to guarantee full activity of the rhodopsin during the infection process. Infected rice seedlings were transferred to pots filled with vermiculite (2–3 mm) and 35 mL H_2_O. Four independent assays were performed with 15 and 12 seedlings/assay for infected (CarO^+^, CarO^−^, ΔOpsA, wild type) and control plants, respectively. To ensure sufficient nutrition of the rice plants, 20 mL 0.5 × modified Rigaud–Puppo (RP) medium [69] were added; RP medium composition (g L^−1^): KNO_3_ 0.5, KH_2_PO_4_ 0.2, K_2_SO_4_ 0.2, MgSO_4_·7H_2_O 0.2, CaCl_2_·2H_2_O 0.1, and 1 mL/L DG microelement solution [65]. Plants were grown at 28 °C in 12/12 light–dark cycles with additional green light. Three days after embedment, plants were fed with 20 mL 0.5× modified RP medium, and 4 days later with 35 mL H_2_O. Plant growth was documented at days 3–7 and 10 after infection by photography. Plant length and internodal distances were determined with the image analysis software ImageJ, version 1.50f [67], and data from 4 assays were pooled for further analysis.

### 4.7. Determination of Chlorophyll and Carotenoid Plant Content

After the experiment, the overall chlorophyll content and the dry weight of the plants were determined. Plants were cut directly at the seed and freeze-dried for 1 day before their dry masses were determined. Plants were pulverized in a mortar and extracted in 80% acetone overnight. The plant debris was removed by centrifugation (1200× *g*, 5 min, 4 °C). The optical density was measured at 663.2 nm and 646.8 nm for chlorophylls, and 470 nm for carotenoids, and their contents were calculated as previously described [70].

### 4.8. Gibberellin Production

For gibberellin measurements, 10^6^ spores of *Fusarium* strains were inoculated in 250 mL of low nitrogen ICI minimal medium [71] with 10% of NH_4_NO_3_ in 500 mL flasks. Cultures were incubated at 30 °C with 200 rpm rotary shaking. After 7 days of growth in the dark or under illumination (3 Wm^−2^), cultures were filtered and centrifuged to get rid of mycelia and spores, respectively. Mycelia were dried at 80 °C for 24 h to measure dry weight, and culture supernatants were kept at −20 °C until measurement of gibberellins. The method used to determine gibberellin was an improvement of a protocol described by Candau et al. [72].

Fluorescent derivatives of gibberellins were obtained by shaking together 50 µL of culture supernatant, 50 µL of ethanol (96%, *v*/*v*), and 500 µL of a cooled mixture of equal volumes of sulfuric acid, and 96% ethanol, and incubating the reaction mixture at 48 °C in 1.5 mL reaction tube for 30 min. After that, 200 µL of each reaction was pipetted in a 96-well plate. Fluorescence was measured in a black microtiter box on a Synergy microplate reader (Biotek, Winooski, VT, USA) at excitation and emission wavelengths of 360 nm and 460 nm. GA3 (Sigma-Aldrich, St. Louis, MO, USA) was used as a standard. Measurements were done from two different experiments, each one with two culture replicates.

### 4.9. Gene Expression Analyses

Cultures consisted in 500 mL flasks with 100 mL of DG media, inoculated with 10^6^ conidia of the wild type strain FKMC1995, and grown for 3 days in an orbital shaker at 150 rpm at 30 °C in the dark. Afterwards, 25 mL of the cultures were transferred to Petri dishes under red safe light and incubated for 8 h in the dark at the same temperature for adaptation to the new conditions. Then, the Petri dishes were used for mycelia collection before or after exposure to white light (7 W m^−2^) for 30, 60, and 240 min. Mycelia from each Petri dish was filtered, frozen in liquid nitrogen, and ground with a mortar and a pestle for RNA extraction with the TRIzol™ (Ambion, Life Technologies, Waltham, MA, USA) reagent. Total RNA concentrations were estimated with a Nanodrop ND-1000 spectrophotometer (Nanodrop Technologies, Wilmington, DE, USA). RNA (2.5 μg) was treated with DNAse (Affymetrix, Thermo Fisher Scientific, Waltham, MA, USA), and reverse transcribed to cDNA with Transcriptor first-strand cDNA synthesis kit (Roche, Mannheim, Germany). Final concentrations were set to 25 ng μL^−1^. qRT-PCR analyses were performed in a LightCycler 480 real-time instrument (Roche) with the LightCycler 480 SYBR green I Master (Roche). Genes and primer sets (forward vs reverse in 5′->3′ orientation) are described in Table 1. Transcript levels for each gene were normalized against the tubulin beta chain gene under the same conditions. Data of three biological replicates, each one with three technical replicates, were averaged.

### 4.10. Sequence Analyses

Protein sequences from the rhodopsins genes identified in 38 fungal genomes were aligned with T-Coffee [73] and used for PhyML [74] analysis with a SH-like and Chi2-based approximate likelihood-ratio test (aLRT) using the LG substitution mode. Phylogenetic tree was generated with the TreeDyn analysis [75]. G proteins in the *F. fujikuroi* genome were identified by protein BLAST analyses through the NCBI server [76] with the sequences of formerly identified G proteins of *Gibberella zeae* [37]. The bootstrap NJ tree was obtained with the ClustalX 1.83 program [77], excluding gaps, and applying the correction for multiple substitutions. The tree was represented with the NJPlot program [78].

## Figures and Tables

**Table 1:** Primers used in the qRT-PCR assays.

Gene	Forward Primer	Reverse Primer
FFUJ_11802 (*carRA*)	CAGAAGCTGTTCCCGAAGACA	TGCGATGCCCATTTCTTGA
FFUJ_11803 (*carB*)	TCGGTGTCGAGTACCGTCTCT	TGCCTTGCCGGTTGCTT
FFUJ_11804 (*carO*)	TGGGCAACGCAGTGACAT	TGCGCAGACAGCCCAGTA
FFUJ_04487	CAACTACCGGCCAACTGTCT	TCTGCATGTGCCTTGTTCTC
FFUJ_05248	TTCGGAAGCTTGCAACAACG	TCGGTGGGTTGATTCGTGAG
FFUJ_06643	CAGCTATCCTGCAGAAGCGA	CATGCTCATCGCCGAAAAGG
FFUJ_07379	TAACCCCGACAACGAGAAAC	GTCTACCCACAGGGCTTTGA
FFUJ_08667	GATGTCCTCCGATCTCGTGT	CTTTCGCTCGGATCTTTGAC
FFUJ_09550 (Gβ type)	ATCACCTCGGTGGCTACATC	ATGTCCCAAACCTTGCACTC
FFUJ_03226 (Gγ type)	ACCGAGCTCAACAATCGTCT	TGCAGTAGGCAATGATGCTC
FFUJ_04397 (tubulin β)	CCGGTGCTGGAAACAACTG	CGAGGACCTGGTCGACAAGT

## Supplementary Materials

Supplementary materials can be found at www.mdpi.com/1422-0067/19/1/215/s1.

## Abbreviations

BLAST	Basic Local Alignment Search Tool
BR	Bacteriorhodopsin
DPSS	Diode Pumped Solid State
GA	Gibberellic Acid
IAA	Indole-3-Acetic Acid
LR	*Leptosphaeria* Rhodopsin
NaAc	Sodium Acetate
NaGlu	Sodium Gluconate
NR	*Neurospora* Rhodopsin
ORP	Opsin Related Protein
SEM	Standard Error of the Mean
TM	Transmembrane Helix
WOA	Weak Organic Acid
