# Supplementary material for: Protein Activity of the Fusarium fujikuroi Rhodopsins CarO and OpsA and Their Relation to Fungus–Plant Interaction
Source: Int J Mol Sci. 2018 Jan 11;19(1):215. doi: 10.3390/ijms19010215 (PMC5796164; doi:10.3390/ijms19010215)
Supplement: Supplementary file 1 [file ijms-19-00215-s001.pdf]

Supplementary Information

Protein activity of the *Fusarium fujikuroi* rhodopsins CarO and OpsA and their relation with fungus-plant interaction

Alexander Adam, Stephan Deimel, Javier Pardo-Medina, Jorge García-Martínez, Tilen Konte, M. Carmen Limón, Javier Avalos, Ulrich Terpitz

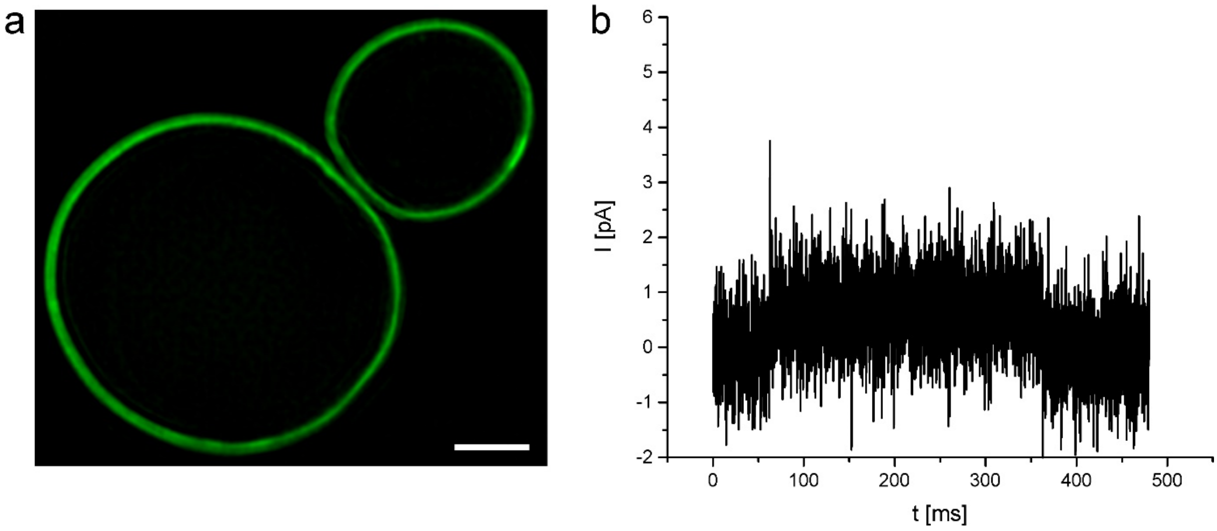

Supplementary Figure S1. Expression of CarO::YFP in baker yeast. a. Confocal image of CarO::YFP localized in the plasma membrane. b. Typical whole cell recording of CarO::YFP pump signal after expression in yeasts.

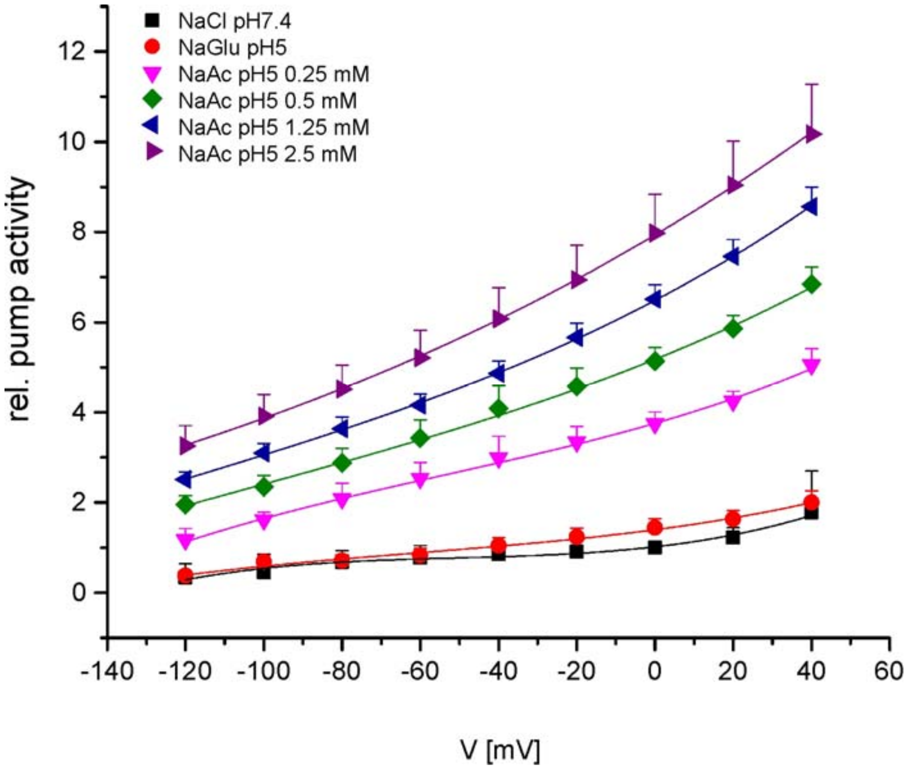

Supplementary Figure S2. Patch-clamp analysis of the influence of IAA in the pump activity of CarO. Current-voltage relationship of the CarO pump activity as indicated in sodium chloride pH 7.4, sodium gluconate pH 5, or various concentration of IAA in sodium gluconate pH 5. Mean + s.e.m. of at least 5 cells are given.

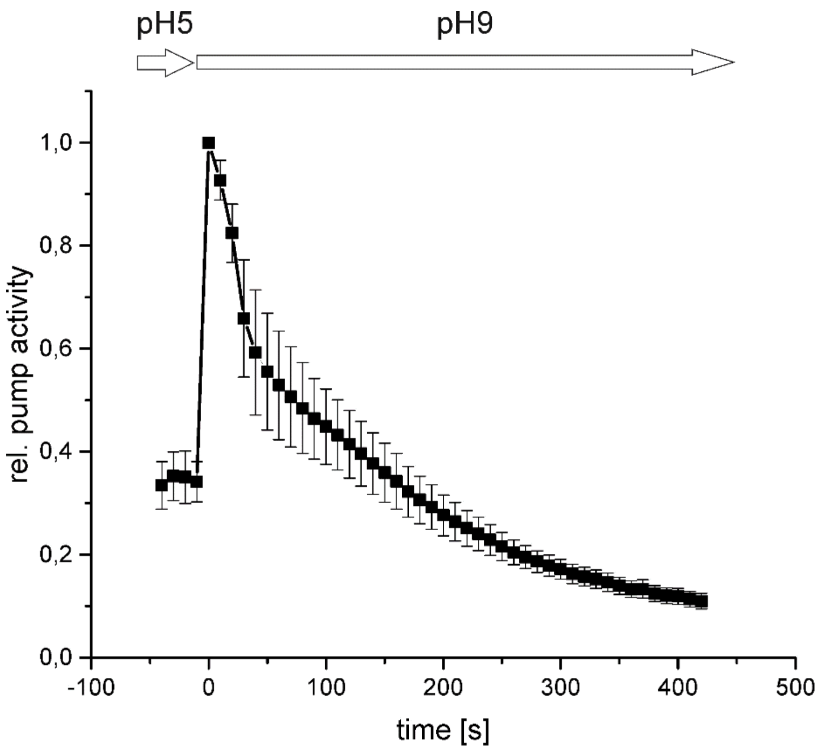

Supplementary Figure S3. Electrophysiological analysis of the relaxation of pump activity of CarO exposed to 0.7 mM sodium acetate after pH jump from 5 to 9. The relative current was measured every 10 s and normalized to the maximal amplitude. After an initial increase by a factor of about 3 the pump intensity decreased below its initial activity at pH5 within several minutes.

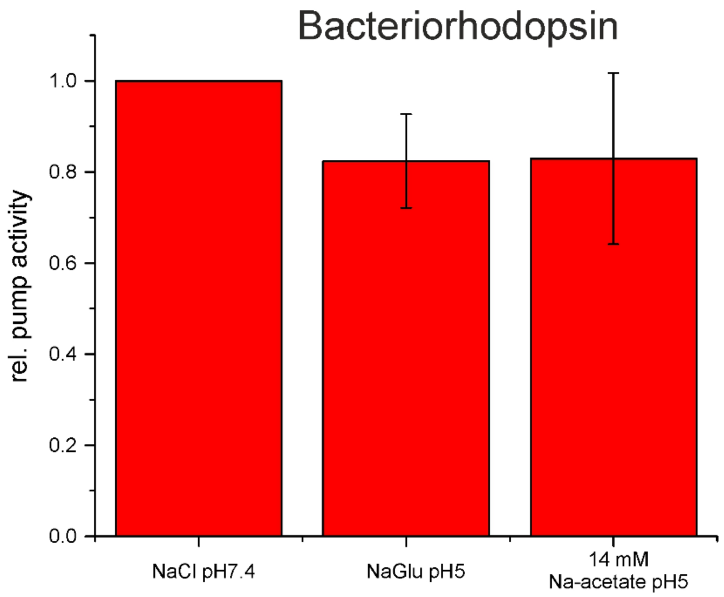

Supplementary Figure S4. Electrophysiological analysis of bacteriorhodopsin (BR::mKateA) expressed in HEK293 cells in the presence and absence of acetate. Upon lowering the pH also the pump activity is slightly reduced due to the higher proton gradient working against the pump. However, the addition of 14 mM does not affect the pump activity of BR.

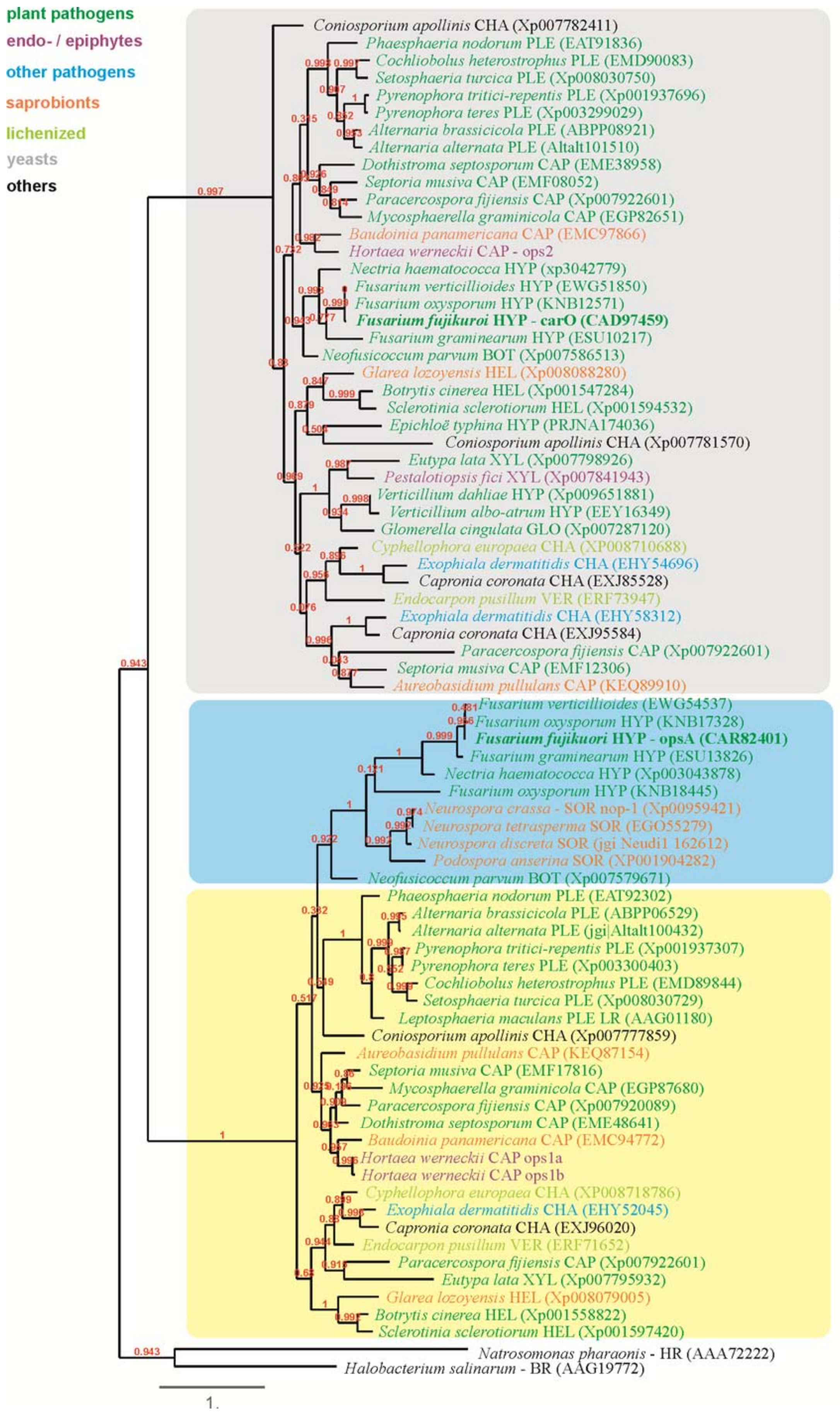

Supplementary Figure S5. Phylogenetic tree of all rhodopsin genes listed in Supplementary Table 1.

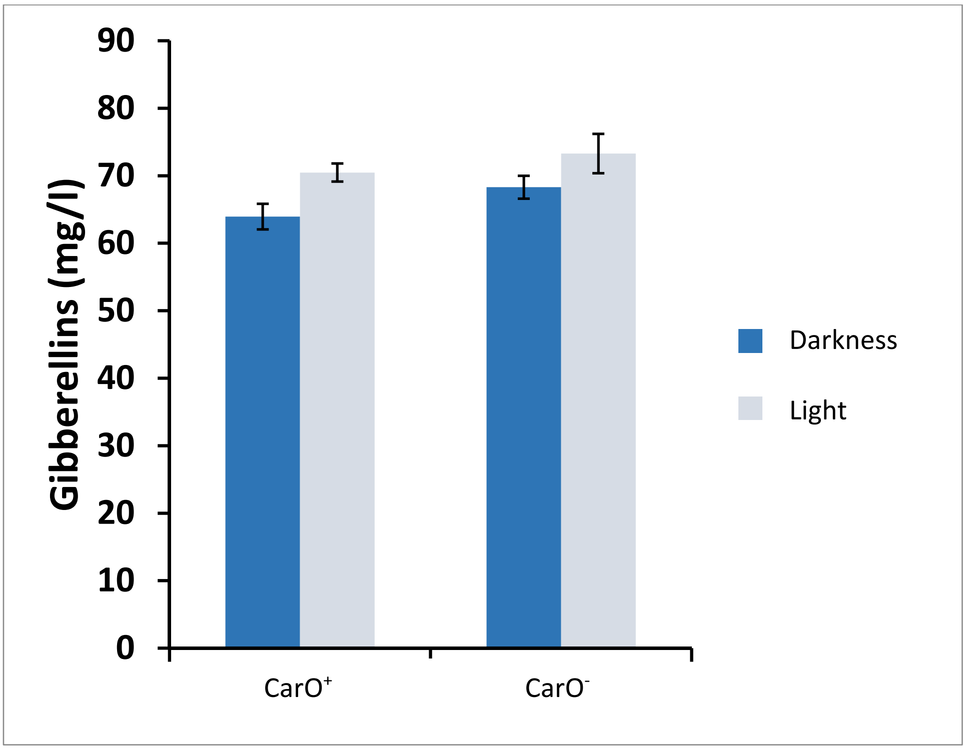

Supplementary Figure S6. Analysis of the gibberellin content in the culture filtrate of either CarO+ or CarO- strain grown either in the dark or the light. No significant change in gibberellin content was observed nor between the strains neither between kind of illumination.

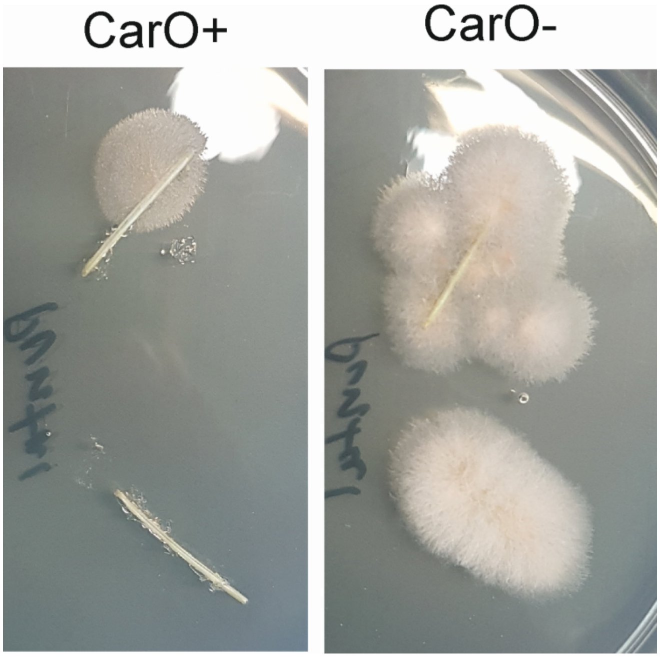

Supplementary Fig. S7. Invasion of fungal strains in rice plants. In this example the cultivar Puntal was chosen, which to our observation is much more susceptive to *F. fujikuroi* infection. The infected plants often exhibit stunted growth or even die. Rice seeds were infected and grown as described for the rice plant infection experiments. On day 7, pieces of about 1 cm length were cut from the stem above the first internode, washed in sterile water and cultivated on PDA Plates by 28°C for 3 days.

Supplementary Table S1. Fungal rhodopsins occurring in genomes of various ascomycetes. Gene accession number for rhodopsins are given.

Class	Order	*Species/Genus*	CarO-like	LR-like	NR-like	plant associated
Sordariomycetes	Hypocreales	*Fusarium verticillioides*	gb|EWG51850	*	gb|EWG54537	yes
		Fusarium oxysporum	gb|KNB12571	*	gb|KNB17328<br>gb|KNB18445	yes
		Fusarium graminearum	gb|ESU10217	*	gb|ESU13826	yes
		Fusarium fujikuroi	gb|CAD97459		gb|CAR82401	yes
		Nectria haematococca	xp003042779	*	xp003043878	yes
		Claviceps purpurea	*	*	*	yes
		Epichloë typhina	PRJNA174036 contig00523 length=26112 numreads=1409	*	*	yes
		Trichoderma reesei	*	*	*	no
		Trichoderma atroviride	*	*	*	no
		Trichoderma virens	*	*	*	no
		Metarhizium anisopliae	*	*	*	no
		Metarhizium acridum	*	*	*	no
		Verticillium dahliae	xp009651881	*	*	yes
		Verticillium albo-atrum	gb|EEY16349	*	*	yes
	Melanosporales	*Melanospora*	*	*	*	no
	Coronophorales	*Bertia*	*	*	*	no
	Microascales		*	*	*	no
	Glomerellales	*Glomerella cingulata*	xp007287120	*	*	yes
	Lulworthiales		*	*	*	no
	Diaporthales	*Togninia minima*	*	*	*	no
	Ophiostomatales	*Papuloso amerospora*	*	*	*	no
		Lanspora coronata	*	*	*	no
	Magnaporthales	*Magnaporthe grisea*	*	*	*	yes
		Gaeumannomyces medullaris	*	*	*	(yes)
	Sordariales	*Neurospora crassa*	*	*	xp959421	no
		Neurospora tetrasperma	*	*	gb|EGO55279	no
		Neurospora discreta	*	*	Neudi1|scaffold_8:371935-372812	no
		Sordaria macrospora	*	*	*	no
		Podospora anserina	*	*	xp001904282	no
	Boliniales		*	*	*	no
	Chaetosphaeriales		*	*	*	no
	Coniochaetales		*	*	*	no
	Xylariales	*Eutypa lata*	xp007798926	xp007795932	*	yes
		Pestalotiopsis fici	xp007841943	xp007831072		yes
Laboulbeniomycetes	Pyxidiophorales		*	*	*	no
	Laboulbeniales		*	*	*	no
Leotiomycetes	Helotiales	*Glarea lozoyensis*	xp008088280	xp008079005	*	no
		Botrytis cinerea	xp001547284	xp001558822	*	yes
		Sclerotinia sclerotiorum	xp001594532	xp001597420	*	yes

		Neobulgaria pura	*	*	*	no
		Pseudeurotium	*	*	*	no
		Bisporella citrina	*	*	*	no
Lecanoromycetes			*	*	*	yes
Eurotiomycetes	Eurotiales	*Aspergillus oryzae*	*	*	*	no
		Aspergillus flavus	*	*	*	no
		Aspergillus terreus	*	*	*	no
		Aspergillus niger	*	*	*	(yes)
		Aspergillus carbonarius	*	*	*	(yes)
		Aspergillus fumigatus	*	*	*	no
		Neosartorya fischeri	*	*	*	no
		Aspergillus clavatus	*	*	*	no
		Aspergilus nidulans	*	*	*	no
		Penicillium chrysogenum	*	*	*	no
		Penicillium marneffei	*	*	*	no
	Coryneliales		*	*	*	(yes)
	Mycocaliciales		*	*	*	no
	Onygenales	*Coccidioides immitis*	*	*	*	no
		Coccidioides posadasii	*	*	*	no
		Uncinocarpus reesii	*	*	*	no
		Trichophyton tonsurans	*	*	*	no
		Trichophyton equinum	*	*	*	no
		Trichophyton rubrum	*	*	*	no
		Arthroderma benhamiae	*	*	*	no
		Trichophyton verrucosum	*	*	*	no
	Chaetothyriales	*Ceramothyrium*	*	*	*	no
		Chaetothyrium	*	*	*	no
		Coniosporium apollinis	xp007781570<br>xp007782411	xp007777859	*	no
		Exophiala dermatitidis	gb|EHY54696<br>gb|EHY58312	gb|EHY52045	*<br>*	no
		Cyphellophora europaea	xp008710688	xp008718786	*	no
		Capronia coronata	gb|EXJ95584<br>gb|EXJ85528	gb|EXJ96020	*	yes
	Verrucariales	*Endocarpon pusillum*	gb|ERF73947	gb|ERF71652	*	yes
		Verrucaria	*	*	*	yes
		Leucocarpia	*	*	*	yes
	Pyrenulales		*	*	*	yea
Arthoniomycetes			*	*	*	
Dothideomycetes	Pleosporales	*Phaeosphaeria nodorum*	gb|EAT91836	gb|EAT92302	*	yes
		Cochliobolus heterostrophus	gb|EMD90083	gb|EMD89844	*	yes
		Setosphaeria turcica	xp008030750	xp008030729	*	yes
		Pyrenophora tritici-repentis	xp001937696	xp001937307	*	yes
		Pyrenophora teres	xp003299029	xp003300403	*	yes
		Alternaria alternata	fgenesh1_kg.1_#_84_#_Locus37v1rpkm2693.34	fgenesh1_pg.1_#_625	*	yes

		Alternaria brassicicola	gb|AB08921	gb|AB06529	*	yes
		Pleospora	*	*	*	yes
		Teichospora	*	*	*	no
	Jahnulales		*	*	*	no
	Patellariales		*	*	*	no
	Capnodiales	*Dothistroma septosporum*	gb|EME38958	gb|EME48641	*	yes
		Mycosphaerella fijiensis	xp007922601<br>xp007927932	xp007920089	*	yes
		Septoria musiva	gb|EMF08052<br>gb|EMF12306	gb|EMF17816	*	yes
		Mycosphaerella graminicola	gb|EGP82651	gb|EGP87680	*	yes
		Baudoinia panamericana	gb|EMC97866	gb|EMC94772	*	no
		Hortaea werneckii	Hwops2	Hwops1a/Hwops1b	*	no
	Myriangiales		*	*	*	yes
	Dothideales	*Aureobasidium pullulans*	gb|KEQ89910	gb|KEQ87154	*	yes
		Hysteropatella	*	*	*	(yes)
		Helicomyces roseus	*	*	*	no
		Venturia inaequalis	*	*	*	yes
		Phaeotrichum benjaminii	*	*	*	no
	Trypetheliales		*	*	*	(yes)
	Botryosphaeriales	*Neofusicoccum parvum*	xp007586513	xp007579671	*	yes
Orbiliomycetes		*Dactylellina*	*	*	*	no
		Arthrobotrys	*	*	*	no
Pezizomycetes			*	*	*	no
Saccharomycetes			*	*	*	no
Taphrinomycetes			*	*	*	yes
Schizosaccharomycetes			*	*	*	no
Pneumocystidiomycetes			*	*	*	no
Neolectomycetes			*	*	*	(yes)

Supplementary Table S2: Expression profile of carotenoid enzymes and G proteins in *in vitro* cultures and plant environment.

IMI58289	FKMC1995	6 mM Gln	*in planta*	ratio
FFUJ_11801 (*carX*)	FFC1_12355	1.1	0.3	0.3
FFUJ_11802 (*carRA*)	FFC1_12354	17.9	19.7	1.1
FFUJ_11803 (*carB*)	FFC1_12353	8.8	23.3	2.7
FFUJ_11804 (*carO*)	FFC1_12352	0.7	2.2	3.2
FFUJ_04487	FFC1_09978	46.4	32.9	0.7
FFUJ_05248	FFC1_13600	5.1	6.5	1.3
FFUJ_06643	FFC1_14186	319.9	302.9	0.9
FFUJ_07379	FFC1_02931	28.2	14.6	0.5
FFUJ_08667	FFC1_14186	7.1	9.8	1.4
FFUJ_09550 (G_b type)	FFC1_08836	195.6	122.6	0.6
FFUJ_03226 (G_g type)	FFC1_07651	431.4	427.0	1.0
FFUJ_04397 (*tubulin b*)	FFC1_09881	127.5	314.2	2.5
FFUJ_02352 (*opsA*)	FFC1_15076	18.0	515.7	28.6

Supplementary Table S3: Clustering of *carO*-like rhodopsins with other car-genes in selected ascomycetes shown in Fig. 3

Fusarium fujikuroi	CarX	CarRA	CarB	CarO	Remarks
Function	Carotenoid oxygenase	phytoene synthase	phytoene dehydrogenase	rhodopsin	
Endorcarpon pusillum	JGI|EPUS_5373	JGI|EPUS_5372	JGI|EPUS_5371	JGI|EPUS_5370	
Aureobasidium pullulans	JGI|286581	JGI|262622	JGI|264275	JGI|350757	*var. pullulans*, strain EXF-150
Epichloe typhina	PRJNA174036 contig00523			PRJNA174036 contig00523	
Fusarium oxysporum	KNB12575	KNB12574 /KNB12573	KNB12572	KNB12571	
Botrytis cinerea	XP_001547287	XP_001547286	XP_001547285	XP_001547284	
Alternaria alternata	JGI|951679	JGI|1026898	JGI|951686	JGI|1015106	strain SRC1lrK2f v1.0
Verticillium dahliae	xp_009651882	-	-	xp_009651881	
Nectria haematococca	xp_003042854	xp_003042777	xp_003042778	xp_003042779	
Neurospora crassa	-	-	-	nop-1	
Leptosphaeria maculans	JGI|6464	JGI|6467	JGI|6465	JGI|6466 / AAG01180	strain JN3
